# Chondroitin Sulfate Disaccharides, a Serum Marker for Primary Serous Epithelial Ovarian Cancer

**DOI:** 10.3390/diagnostics11071143

**Published:** 2021-06-23

**Authors:** Karina Biskup, Caroline Stellmach, Elena Ioana Braicu, Jalid Sehouli, Véronique Blanchard

**Affiliations:** 1 Institute of Laboratory Medicine, Clinical Chemistry and Pathobiochemistry, Charité-Universitätsmedizin Berlin, Corporate Member of Freie Universität Berlin, Humboldt-Universität zu Berlin, and Berlin Institute of Health, 13353 Berlin, Germany; karina.biskup@charite.de (K.B.); caroline.stellmach@charite.de (C.S.); 2 European Competence Center for Ovarian Cancer, Department of Gynecology, Berlin Institute of Health, Charité-Universitätsmedizin Berlin, Corporate Member of Freie Universität Berlin, Humboldt-Universität zu Berlin, 13353 Berlin, Germany; elena.braicu@charite.de (E.I.B.); jalid.sehouli@charite.de (J.S.)

**Keywords:** chondroitin sulfate, disaccharide, glycosaminoglycan, ovarian cancer, biomarker, HPLC

## Abstract

Glycosaminoglycans are long polysaccharidic chains, which are mostly present in connective tissues. Modified GAG expression in tissues surrounding malignant cells has been shown to contribute to tumor progression, aggressive status and metastasis in many types of cancer. Ovarian cancer is one of the most lethal gynecological malignancies due to its late diagnosis because of the absence of clear symptoms and unavailability of early disease markers. We investigated for the first time GAG changes at the molecular level as a novel biomarker for primary epithelial ovarian cancer. To this end, serum of a cohort of 68 samples was digested with chondroitinase ABC, which releases chondroitin sulfate into disaccharides. After labeling and purification, they were measured by HPLC, yielding a profile of eight disaccharides. We proposed a novel GAG-based score named “CS- bio” from the measured abundance of disaccharides present that were of statistical relevance. CS-bio’s performance was compared with CA125, the clinically used serum tumor marker in routine diagnostics. CS-bio had a better sensitivity and specificity than CA125. It was more apt in differentiating early-stage patients from healthy controls, which is of high interest for oncologists.

## 1. Introduction

Although surgical techniques and therapies have improved in the past years, ovarian cancer (OC), one of the rarer gynecological carcinoma types, remains the most lethal gynecological malignancy [1]. The reason for the high mortality rate is that the disease remains clinically silent in the early stages and tends to be diagnosed only when the late stages are reached [2]. Hence, only 10–20% of patients are diagnosed in the early stages (stage I: localized in the ovary, stage II: localized in the pelvis) whereas approx. 80% of patients are diagnosed at a late stage of the disease (stages III and IV) [3,4]. For decades, the standard of care has been debulking surgery followed by platinum- or taxane-based chemotherapy. Prognosis directly correlates with the presence and size of residual tumors remaining after surgery. Indeed, most patients initially respond to chemotherapy but will become chemo-resistant upon relapse [5]. As a result, insufficient screening methods and late-stage detection are the primary causes of poor prognosis. OC is diagnosed using pelvic examination and transvaginal ultrasound in combination with the measurement of the serum levels of Carbohydrate Antigen 125 (CA125). Regarding primary diagnosis, CA125 alone has a low sensitivity of 57%, especially in early stages of OC and a specificity of 97% [6]. The other routine biomarker available is Human Epididymis 4 (HE4). It shows slightly better performance than CA125 in pre-menopausal women but has otherwise low sensitivity [7]. Modeling simulations estimated that early diagnosis could not only improve survival by 10–30% but also be cost-effective [8].

As glycosylation is modulated at the onset and the course of cancer, it is an interesting source of biomarkers [9,10,11,12]. Glycosaminoglycans (GAG) are glycoconjugates, which are mostly present in connective tissues. They are found either in free form or as constituents of proteoglycans in the extracellular matrix [13]. Being composed of long polysaccharidic chains, GAG are key components of the extracellular matrix in healthy connective tissues and play an important role in homeostasis, regulation of cell growth, angiogenesis, migration, recognition and disease [14]. Due to their structural diversity, GAG actively take part in cellular events by binding a wide range of different molecules (for instance growth factors, cytokines, chemokines, enzymes) at the surface of cells and tissues. As a consequence, GAG promote inflammation that accompanies many types of inherited and acquired diseases. GAG modulations are observed during the development of many diseases (cancer, neuroinflammation, atherosclerosis, diabetes) within tissues and, as a result, in blood that is in circulation [15,16].

Biological processing of glycoconjugates unlike proteins is not template-driven and results from the availability of activated monosaccharides, glycosidases, glycosyltransferases, sulfotransferases and epimerases. GAG are negatively charged linear polysaccharide chains that are built of disaccharide repeating units. This study concentrates on the analysis of non-sulfated hyaluronic acid (HA)—consisting of β(1-3)-GlcNAcβ(1-4)-GlcA—as well as chondroitin sulfate (CS)—consisting of β(1-3)-GalNAcβ(1-4)-GlcA. CS-0S denotes a non-sulfated disaccharide. CS sulfation can occur at the C2 position of glucuronic acid (GlcA), (named CS-2S) and the C4 and C6 position of *N*-acetylgalactosamine (GalNAc), named CS-4S or CS-6S. Combinations of these sulfation patterns are possible, resulting in the disaccharides CS-2S4S, CS-2S6S, CS-4S6S, CS-2S4S6S.

Modified GAG expression in tissues surrounding malignant cells has been shown to contribute to tumor progression, aggressive status and metastasis for many types of cancer, namely ovarian cancer [17], breast cancer [18], colorectal cancer [19], hepatocellular carcinoma [20], renal cell carcinoma [21], pancreatic cancer [22], and gastric carcinoma [23]. In addition, elevated levels of chondroitin synthase I and III, which synthesize CS chains, were measured in various types of cancer [24,25,26,27]. CS chains interact with growth factors or are stored in the extracellular matrix and released gradually within the matrix, promoting cell signaling [16]. In other words, CS overexpression as well as modulated sulfation are hallmarks of cancer development [28,29]. Moreover, CS-proteoglycan expression has been shown to correlate with both tumor differentiation and prognosis [30]. In this work, we investigated for the first time GAG changes at the molecular level as a novel biomarker for primary epithelial ovarian cancer.

## 2. Materials and Methods

All chemicals were purchased from Sigma-Aldrich (St. Louis, MO) unless stated otherwise. MilliQ water was used from a MilliQ Plus Millipore system (Darmstadt, Germany).

### 2.1. Patient Samples

Primary ovarian serum samples were obtained from the Tumor Bank Ovarian Cancer project (http://www.toc-network.de/) of the Charité Medical University (Berlin, Germany). The Ethics Committee approved the use of the samples (EA4/073/06). The patient’s informed consent was obtained prior to surgery or during subsequent treatment. Blood samples were collected prior to surgery. The tumor pattern was intraoperatively prospectively assessed based on the surgical procedures performed, through a systematic interview of the surgical team and by histology. All findings and associated data were registered in a validated documentation system specifically developed for ovarian neoplasms [31,32,33,34,35]. The menopausal status was not provided. Enrolled patients did not suffer from any other forms of cancer. Sepsis samples were obtained as approved by the ethical approval (EA1/285/09) of the Charité Medical University (Berlin, Germany).

In this study, 28 primary serous patients (FIGO: stage 1, *n* = 13; stage 2, *n* = 5; stage 3, *n* = 5; stage 4, *n* = 3; not defined, *n* = 2), 10 patients suffering from sepsis and 30 age-matched healthy controls were enrolled (5 patients suffering from benign ovarian diseases and 25 healthy controls). Patient’s data are presented in Table 1. Blood was collected using serum tubes containing clotting activators (Vacutainer, BD, Medical-Pharmaceutical System, Franklin Lakes, NJ, USA). Collected blood was left to clot at room temperature for 30 min to 2 h, then serum was separated by centrifugation at 1200 g for 15 min. Serum was aliquoted and stored at −80 °C until the time of analysis.

### 2.2. Protein Release

An amount of 50 µL pronase E (20 mg/mL) dissolved in 20 mM CaCl_2_ was added to 50 µL serum aliquots. After adding 150 µL milli-Q water, samples were incubated at 55 °C overnight to achieve protein digestion. On the following day, the reaction was stopped by heating the samples at 95 °C for 10 min. After cooling down, samples were centrifuged at 10,000× *g* for 10 min at 4 °C. Supernatants were applied to 3 kDa Amicon columns.

### 2.3. Release of Disaccharide GAG

Amounts of 5 µL Chondroitinase ABC from *Proteus vulgaris* (10 mU/µL dissolved in 0.01% (wt/vol) BSA) and 200 µL of 200 mM NaOAc, 8 mM CaCl_2_ pH 7.5 were added to the retentates and the total volume was brought to 800 µL with milli-Q water. Samples were incubated overnight at 37 °C at 150 rpm. The following day, they were desalted using 3 kDa Amicon filters following the manufacturer’s instructions. Chondroitinase ABC degrades CS via ß-elimination of internal GlcA residues, yielding an unsaturation denoted ∆.

### 2.4. 2-AB-Labeling and Purification

Dried GAGs were dissolved in 25 µL 10% acetic acid/milli-Q water solution. They were then mixed with 25 µL labeling solution consisting of 1 M 2-aminobenzamide (2-AB) and 0.24 M 2-picoline-borane in 10% acetic acid/MeOH. The reaction mixture was incubated in a Thermomixer for 3 h at 50 °C, shaken at 500 rpm in the dark. The reaction was stopped by the addition of 50 µL of milli-Q water.

### 2.5. Purification of 2-AB-Disaccharides Using G10 Columns

PD MiniTrap G10 columns (GE Healthcare, Solingen, Germany) were equilibrated with 6 column volumes of milli-Q water. Labeled samples, brought to a volume of 100 µL using Milli-Q water, were loaded onto the columns, which were subsequently washed with 550 µL milli-Q water. Samples were eluted with 1250 µL milli-Q water and dried under vacuum.

### 2.6. HPLC Measurements

The 2-AB-labeled GAG disaccharides were dissolved in 90% ACN then measured by HPLC (Dionex, Idstein, Germany) using a SeQuant ZIC-HILIC column (Merck Millipore, Germany) and detected by fluorescence (λ_excitation_ = 330 nm and λ_emission_ = 420 nm). Separations were performed at 25 °C with a gradient elution at a flow rate of 200 μL/min for 95 min using H_2_O (solvent A), acetonitrile (ACN, solvent B) and 200 mM ammonium acetate buffer (NH_4_OAc, solvent C). The applied gradient was initiated with 5% A, 90% B, 5% C then was increased in 60 min to 30% A, 65% B, 5% C. Peaks were assigned by comparison with a standard mixture of the following 9 disaccharide standards: ∆HexA(β1-3)GlcNAc (HA-0S), ∆HexA(β1-3)GalNAc (CS-0S), ∆HexA2*S*(β1-3)GalNAc (CS-2S (Dextra, Reading, UK)), ∆HexA(β1-3)GalNAc4*S* (CS-4S), ∆HexA(β1-3)GalNAc6*S* (CS-6S (Dextra, Reading, UK)), ∆HexA2*S*(β1-3)GalNAc6*S* (CS-2S6S (Dextra, UK)), ∆HexA2*S*(β1-3)GalNAc4*S* (CS-2S4S (Dextra, UK)), ∆HexA(β1-3)GalNAc4*S*6*S* (CS-4S6S (Dextra, Reading, UK)), ∆HexA2*S*(β1-3)GalNAc4*S*6*S* (CS-2S4S6S (Dextra, Reading, UK)). Relative quantification was conducted using the open source OPENchrom software Community Edition 1.3.0. (Hamburg, Germany).

### 2.7. Statistical Analysis

Statistical analysis was performed using SPSS version 25.0 (SPSS Inc., Chicago, IL, USA).

## 3. Results

**Figure 1:**
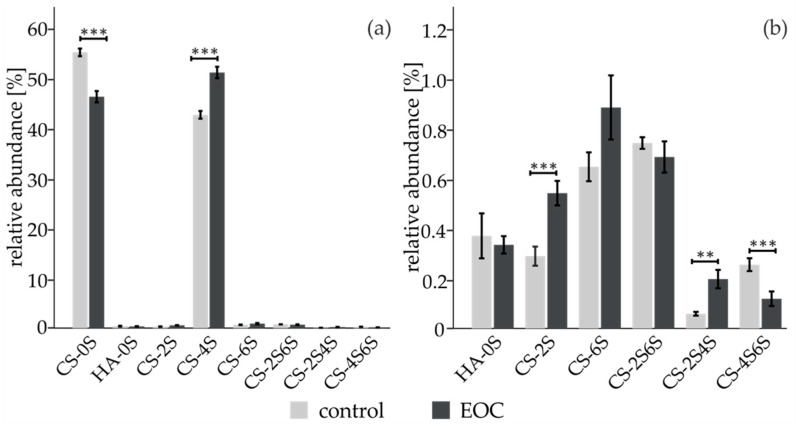
(**a**) Mean and 95% confidence intervals [1] of relative areas of CS disaccharides released from human serum measured by HPLC coupled to fluorescence detection. (**b**) Enlarged view of the low abundant CS disaccharides. * *p* < 0.5, ** *p* < 0.01, *** *p* < 0.001 as judged from non-parametric Mann–Whitney U-test. S indicates sulfate and the preceding digit indicates the sulfation position.

We analyzed the serum CS- content of 28 primary ovarian cancer patients, 10 sepsis patients and 30 age-matched healthy volunteers. To do so, the protein moiety of proteoglycans was first cleaved using pronase, a cocktail of proteases. After purification, the resulting glycosaminoglycan samples were digested with chondroitinase ABC yielding CS disaccharides as well as the hyaluronic acid disaccharide (HA). They were subsequently labeled with 2-AB, a neutral fluorescent label, then measured by HPLC using a HILIC column. Representative spectra are presented in Supplementary Figure S1. Repeatability of HPLC measurements and of sample preparation is presented in Supplementary Tables S1 and S2, respectively. The one-sample Shapiro–Wilk test was carried out to assess the distribution normality in each group (healthy controls, early stage = FIGO I and FIGO II, late stage = FIGO III + FIGO IV). It showed that the data was not normally distributed and therefore non-parametric tests were used for further statistical evaluation. Bar charts were generated to visualize the distribution of relative areas of single GAG-structures between EOC serum and the healthy control group (Figure 1). Error bars are shown as standard errors [36]. A Mann–Whitney U-test was performed to assess the statistical significance between EOC patients and controls. *p*-Values smaller than 0.05 were considered to be statistically significant. CS-0S and CS-4S are the two most abundant structures and constitute the majority of the CS disaccharide pool (Figure 1a). The six disaccharides HA-0S, CS-2S, CS-6S, CS-2S6S, CS-2S4S and CS-4S6S were detected in small amounts but the trisulfated disaccharide CS-2S4S6S was not present in serum samples.

Statistically significant increases of the monosulfated CS-2S, CS-4S and the disulfated CS-2S4S were observed accompanied by statistically significant decreases of the non-sulfated CS-0S and the disulfated CS-4S6S (Figure 1b). Heparin and heparan sulfate from human serum were also isolated and then further digested with a combination of heparinase I, II and III but no statistically significant changes between EOC patients and controls were observed (data not shown). In order to address whether the observed changes of CS-disaccharides in EOC patients were related to the inflammation that accompanies cancer development, we added a cohort of 10 patients suffering from sepsis Supplementary (Figure S2) to this work. The CS-disaccharidic profile of sepsis patients is very different from the profile of EOC patients.

Thereafter, receiver operating characteristic (ROC) curves were built for the CS disaccharides that were of statistical relevance (Figure S3). The greatest areas under the curve (AUC) were obtained for CS-0S (0.81), CS-2S (0.88), CS-4S (0.74) and CS-2S4S (0.91). Next, we tested which combinations of these CS disaccharides could improve ovarian cancer diagnostics using binary logistic regression. The following prediction model, named „CS-bio“, was built: 27.974 − 0.642×CS-0S + 4.633×CS-2S + 27,205×CS-2S4S. The logistic regression model showed statistical significance [χ^2^_(3)_ = 60.301, *p* < 0.001], as it explained 86.2% (Nagelkerke R^2^) of the variance in ovarian cancer.

Few publications have addressed age-dependency of circulating GAG. Hence, we investigated whether a correlation could be established between the CS-disaccharide species that make up the CS-bio marker and the age of ovarian cancer patients and healthy controls using a linear regression model. Linear graphs with the best fitting regression lines are illustrated with a scatter point diagram (Figure S4). In addition, coefficients of determination (R^2^), *β* regression coefficients and *p*-values were calculated. We found an absence of correlation between age and abundance of CS disaccharides (Figure S4). Therefore, we concluded that presence of CS-0S, CS-2S and CS-2S4S was independent of the age of patients and controls.

**Figure 2:**
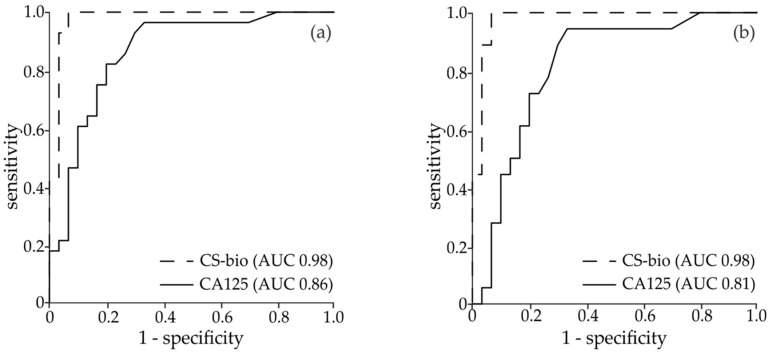
ROC curves of the biomarkers CS-bio and CA125 for (**a**) all EOC (*n* = 26) and control samples included in this study (*n* = 30) and (**b**) early stage (FIGO I + II, *n* = 18) and control samples (*n* = 30).

Binary logistic regression was carried out to further evaluate the ability of the diagnostic GAG-based marker to correctly classify the cohort of patients. The diagnostic accuracy of CA125 and CS-bio was assessed by building the ROC curve at 95% confidence interval (CI) and calculating the AUC. CS-bio had an AUC of 0.98, which was superior to CA125 (0.87) for both the whole cohort of patients and early-stage patients only (AUC: 0.87 (Figure 2a) and 0.81 (Figure 2b), respectively). Overall, CS-bio had a better sensitivity (100%) and specificity (93%) than CA125 (60% and 83% respectively).

**Figure 3:**
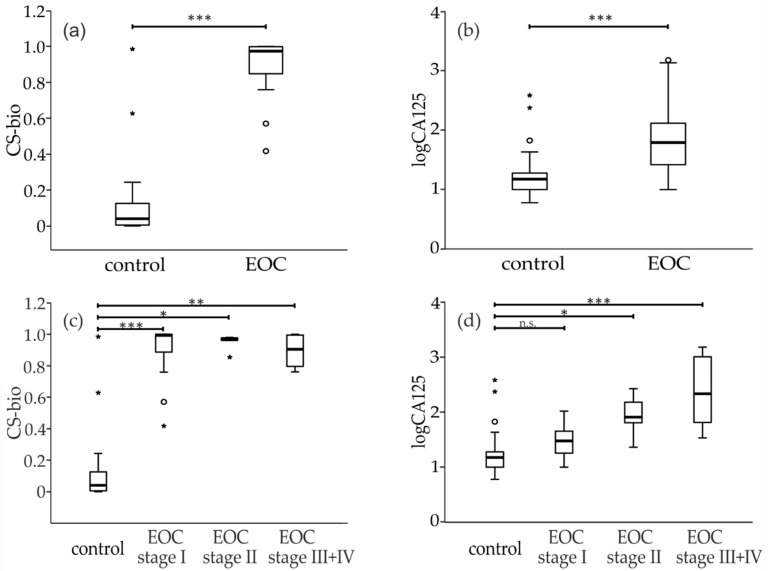
Boxplots were performed for CS-bio and logarithmic values of CA125. In (**a**,**b**), the whole cohort was considered in the EOC group. In (**c**,**d**), the EOC cohort was divided according to stages. The Bonferroni corrected *p*-values (* *p* < 0.5, ** *p* < 0.01, *** *p* < 0.001, n.s. non-significant) were estimated from non-parametric multiple pairwise comparison Jonckh eere–Terpstra test (T_JT_).

Box plots were generated to examine the subcategories of patients (Figure 3). All box plots were created after logarithmic transformation of CA125 values, which reduces skewness in the distribution of the results. Independent variables consisted of four ordinal groups of cancer progression (healthy controls, FIGO I, FIGO II and late stage = FIGO III + FIGO IV), while dependent variables were measured on a continuous level. A non-parametric multiple comparison Jonckheere–Terpstra test (T_JT_) was carried out to test the significance during cancer progression. *p*-Values were calculated after Bonferroni correction and are presented on Figure 3. Both CS-bio and CA-125 were able to discriminate between healthy controls and EOC patients (Figure 3a,b). Interestingly, CS-bio was statistically upregulated in early-stage patients (FIGO I) whereas CA125 was not (Figure 3c,d).

## 4. Discussion

In this work, we analyzed the CS-glycosaminoglycome from the serum of primary epithelial ovarian cancer patients and compared it with age-matched healthy controls. Seven CS disaccharides and the HA disaccharide could be detected. Regardless of the analyzed disease states, CS-0S and CS-4S disaccharides were the major structures present in human serum with a mean relative abundance of around 50–65% and 33–49% respectively. The six other disaccharides, namely HA-0S, CS-2S, CS-6S, CS-2S4S, CS-2S6S and CS-4S6S, had low intensities. In addition, no trisulfated CS structures were detected in any of the sera as was observed previously [37]. When compared with mouse serum, human serum contains more non-sulfated CS disaccharides and less disulfated disaccharides [37]. Our data was combined into a ratio named “CS-bio”, which showed a statistically significant elevation in FIGO stage I patients (*p* < 0.001) where CA125 was not different. Interestingly, CS-bio did not increase with the disease stage as CA125 did.

As for other types of glycosylation such as IgG asialylation [12], absolute CS amounts present in human plasma were previously shown to increase with age [38]. In this study, we found that the relative amounts of CS disaccharides in human serum were not affected by age. They remained fairly stable with age, which is in line with findings for renal cell carcinoma [39]. The same was observed previously for the chondroitin sulfate sulfotransferases CHST11 and CHST15 in ovarian cancer patients [40]. Carbohydrate sulfotransferase CHST11 was shown to be elevated in ovarian cancer patients in tumor tissues irrespective of the histological subtype, FIGO stage or post-operative residual tumors [40]. CHST11 is responsible for the formation of CS-4S via the transfer of sulfate to the hydroxyl group at position 4 of the GalNAc. This is in line with the increase of CS-4S and the decrease of CS-0S that were measured in our study as early as in FIGO stage I patients. Traces of CS-6S observed in serum most likely stems from the degradation of plasma cells [41].

It was previously shown using immunohistochemistry and a specific antibody that CS disulfation, particularly CS-4S6S, is enhanced in ovarian cancer tissues [17,30,42] and in human blood [43]. These observations also correlate with increased mRNA expression of GalNAc4S-6-sulfotransferase, the enzyme that is responsible for the attachment of sulfate groups at positions 4 and 6 in CS polysaccharides [44] and with the increased genomic expression of the CS proteoglycan versican [45]. In addition, versican was previously shown to be increased in malignant stroma surroundings and promoted the growth of several forms of cancer including ovarian cancer [46,47,48]. Moreover, it correlated with cancer relapse, metastasis and unfavorable prognosis [49,50] but the detailed CS composition of versican has not yet been studied in detail. With the sample preparation protocol that we used, CS-4S6S was observed only as a minor compound both in controls and in EOC patients. It cannot be ruled out that CS-4S6S could be enriched using another protocol whereas CS-0S could be somehow depleted, for instance in an anion-exchange chromatography step.

The modulation of CS disaccharides was also studied previously in the plasma and urine of renal cell carcinoma patients [39,51,52] but not for serum, which may contain a different profile from plasma as clotting factors are not present. The CS profiles obtained for renal cell carcinoma were different from the ones obtained for EOC in this study: CS- 6S and the ratio CS-6S/CS-4S were increased in renal cancer whereas CS-4S was increased in our study [39,51,52].

One of the proteoglycans involved could be the serine protease inhibitor bikunin, the most abundant proteoglycan present in blood. Synthesized by hepatocytes, the CS moiety of bikunin contains the two motifs CS-0S and CS-4S and is only O-sulfated at the first five GalNAc of the reducing end [53]. Bikunin was previously shown to be overexpressed in the blood and within tumors of ovarian cancer patients [54]. In addition, it was also observed in the secretome of other carcinoma cell lines, such as squamous cell carcinoma, glioma, and rhabdomyosarcoma [55].

## 5. Conclusions

To conclude, we studied for the first time the composition of GAG at the disaccharide level and established a novel serum GAG-based biomarker named CS-bio that was particularly able to differentiate EOC early stages from healthy controls and which outperformed CA125. In future studies, these performances would need to be validated in a larger cohort.

## Figures and Tables

**Table 1:** Demographics of the cohorts used in this study.

	Control	EOC
**No of Patients**	30	28
**Age**		
**mean**	52.6	60
**median**	50.5	59.5
**range**	17–81	35–79
**SD**	13.1	10.9
**stage**		
**I**		13
**II**		5
**III**		5
**IV**		3
**n.d.**		2
**CA125 in kU/L**		
**mean**	36.8	218.5
**median**	15	62
**range**	6–386	10–1504
**SD**	78.3	388

## Supplementary Materials

The following are available online at https://www.mdpi.com/article/10.3390/diagnostics11071143/s1, Figure S1: Representative HPLC chromatograms of 2AB-labeled CS-disaccharides isolated from an ovarian cancer patient (upper panel) and a healthy control (lower panel), Figure S2: (a) Mean and 95% confidence intervals [1] of relative areas of CS disaccharides released from human serum measured by HPLC coupled to fluorescence detection. (b) Enlarged view of the low abundant CS disaccharides. S indicates sulfate and the preceding digit indicates the sulfation position, Figure S3: ROC curves of the disaccharides that were of statistical significance in this study, Figure S4: Association between disaccharide CS-0S (a), CS-2S (b) and CS-4S (c) (Y-axis) and age of the patient cohort (X-axis). Descriptive statistics are in terms of R^2^ (coefficient of determination), b (regression b coefficient) and *p* (*p*-values), Table S1: CS-disaccharides were released from a healthy control, labeled with 2-AB and measured by HPLC in triplicate. Mean, standard deviation and coefficient of variation are presented in the Table for each CS disaccharide, Table S2: CS-disaccharides were released from a healthy control in triplicate, labeled with 2-AB and measured by HPLC. Mean, standard deviation and coefficient of variation are presented in the Table for each CS disaccharide.
