# Supplementary material for: Chondroitin Sulfate Disaccharides, a Serum Marker for Primary Serous Epithelial Ovarian Cancer
Source: Diagnostics (Basel). 2021 Jun 23;11(7):1143. doi: 10.3390/diagnostics11071143 (PMC8304809; doi:10.3390/diagnostics11071143)
Supplement: Supplementary file 1 [file diagnostics-11-01143-s001.zip › diagnostics-1267340-supplementary.pdf]

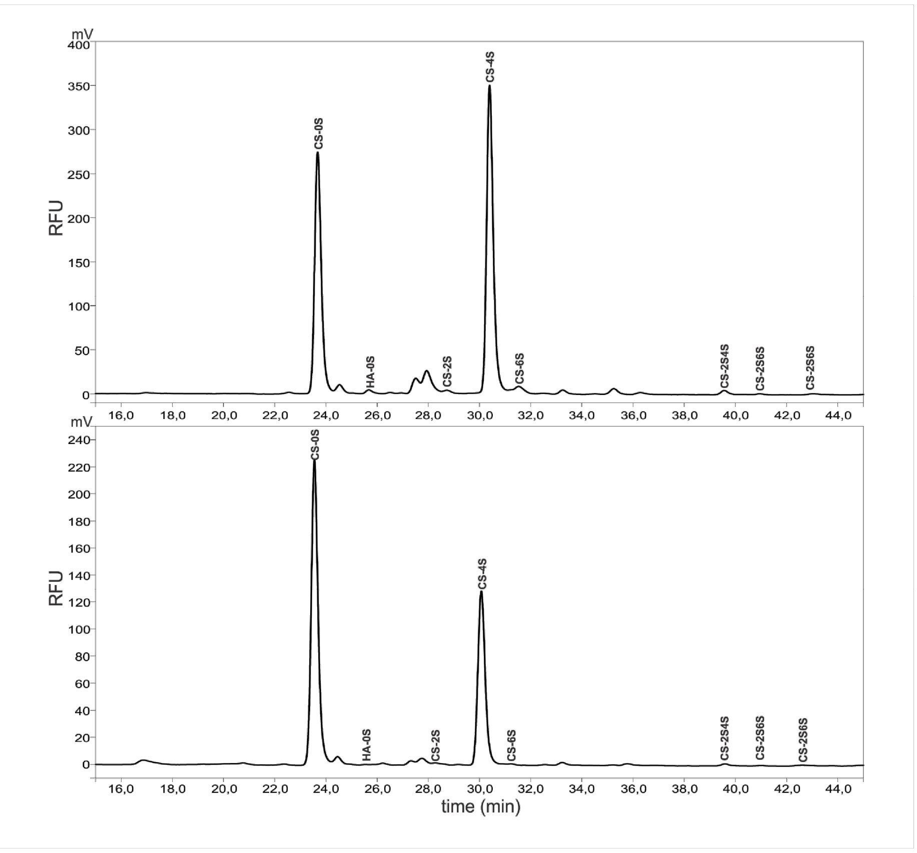

Figure S1. Representative HPLC chromatograms of 2AB-labeled CS-disaccharides isolated from an ovarian cancer patient (upper panel) and a healthy control (lower panel).

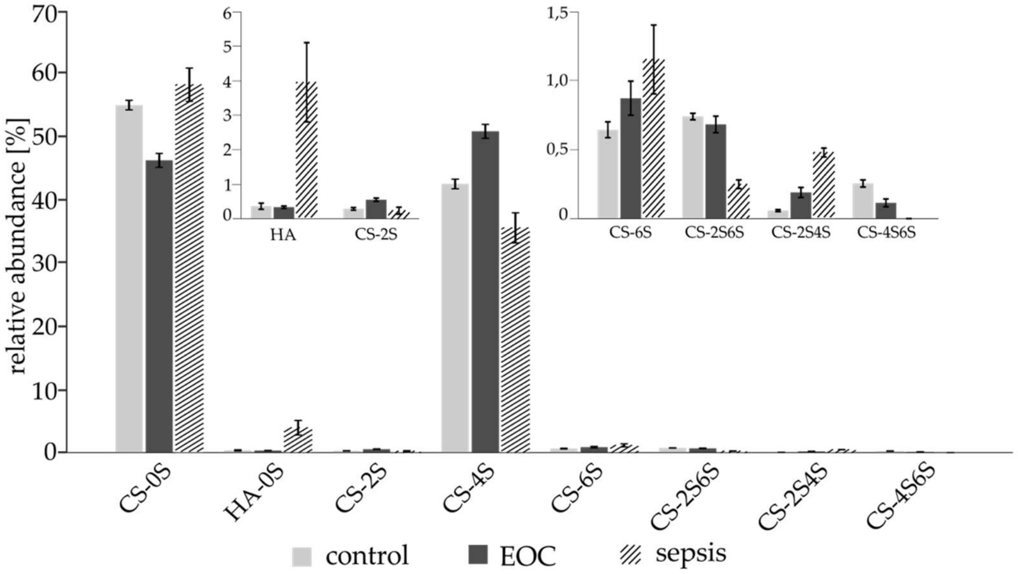

Figure S2. (**a**) Mean and 95% confidence intervals [1] of relative areas of CS disaccharides released from human serum measured by HPLC coupled to fluorescence detection. (**b**) Enlarged view of the low abundant CS disaccharides. S indicates sulfate and the preceding digit indicates the sulfation position.

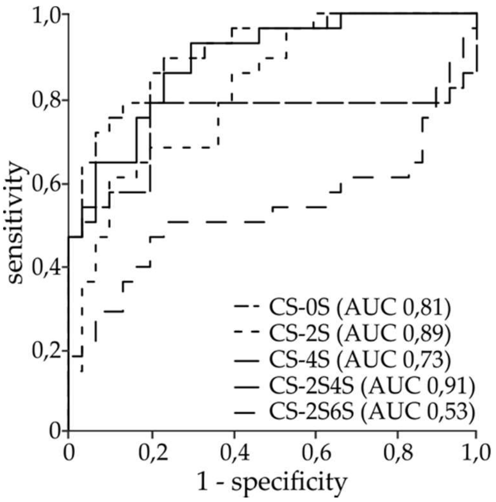

Figure S3. ROC curves of the disaccharides that were of statistical significance in this study.

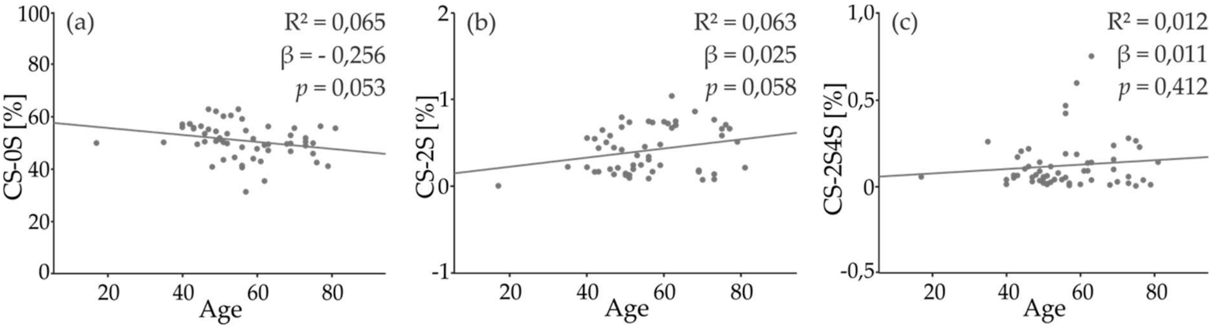

Figure S4. Association between disaccharide CS-0S (**a**), CS-2S (**b**) and CS-4S (**c**) (Y-axis) and age of the whole patient cohort (X-axis). Descriptive statistics are in terms of R2 (coefficient of determination), b (regression b coefficient) and p (p-values).

Supplementary Tables

Table S1. CS-disaccharides were released from a healthy control, labeled with 2-AB and measured by HPLC in triplicate. Mean, standard deviation and coefficient of variation are presented in the Table for each CS disaccharide.

Composition	Mean	Standard Deviation	Coefficient of variation (%)
CS-0S	38,375	0,034	0,09
HA	0,369	0,009	2,30
CS-2S	1,731	0,037	2,16
CS-4S	54,728	0,077	0,14
CS-6S	1,742	0,083	4,74
CS-2S6S	1,857	0,023	1,25
CS-2S4S	0,227	0,010	4,49
CS-4S6S	0,972	0,010	1,04

Table S2. CS-disaccharides were released from a healthy control in triplicate, labeled with 2-AB and measured by HPLC. Mean, standard deviation and coefficient of variation are presented in the Table for each CS disaccharide.

Composition	Mean	Standard Deviation	Coefficient of variation (%)
CS-0S	39,267	0,777	1,98
HA	0,343	0,004	1,24
CS-2S	1,744	0,148	8,47
CS-4S	54,046	0,708	1,31
CS-6S	1,588	0,128	8,09
CS-2S6S	1,814	0,093	5,15
CS-2S4S	0,216	0,015	6,95
CS-4S6S	0,981	0,063	6,37
